# The Effect of Childhood Emotional Adversity and Later Life Friend Solidarity on Cognition

**DOI:** 10.1093/geroni/igab046.2656

**Published:** 2021-12-17

**Authors:** Kaleena Odd, Julie Blaskewicz Boron, Nicholas Turiano, Jonathan Santo

**Affiliations:** 1 University of Nebraska Omaha, Omaha, Nebraska, United States; 2 West Virginia University, Morgantown, Virginia, United States

## Abstract

Early life experiences can influence later life outcomes such as physical, mental, and cognitive health. Previous research investigated the effect of childhood socioeconomic status in relation to mid-to-later life cognition (Liu & Lachman, 2019); however, the effects of childhood emotional adversity on cognition have not been examined. Controlling for age, education, sex, and race, the current study investigated the influence of childhood emotional adversity and later life friend solidarity on change in later life episodic memory, executive functioning, and subjective memory (i.e., perceived memory compared to others same age). Utilizing the Midlife in the United States (MIDUS) database, we studied 2,752 participants (50-75 years, M=60.09, SD=6.97, 53% female, 84% White) with completed measures on MIDUS 1 retrospective childhood adversity, MIDUS 2 friend solidarity, and MIDUS 2/3 cognition. Multilevel modeling (Mplus) was used. Higher friend solidarity was associated with higher executive functioning (b=0.122, p<.01) and higher subjective memory (b=0.267, p<.001), suggesting the positive impact of supportive friendships. Higher childhood emotional adversity was associated with lower perceived subjective memory (b=-0.037, p<.05). There was no significant friend solidarity by emotional adversity interaction. Together, these findings suggest that later life friend solidarity may be important for objective and subjective cognition; whereas, childhood emotional adversity may play a role in subjective cognition. Given the associations in prior research between lower perceptions of memory and lower mental well-being and quality of daily life, experiencing childhood emotional adversity may increase risk of lower perceptions of well-being, including cognitive functioning.

